# Second language experience modulates word retrieval effort in bilinguals: evidence from pupillometry

**DOI:** 10.3389/fpsyg.2014.00137

**Published:** 2014-02-21

**Authors:** Jens Schmidtke

**Affiliations:** Program of Second Language Studies, College of Arts and Letters, Michigan State UniversityEast Lansing, MI, USA

**Keywords:** spoken word recognition, pupillometry, word frequency effect, bilingualism, lexical retrieval, neighborhood density, visual world paradigm

## Abstract

Bilingual speakers often have less language experience compared to monolinguals as a result of speaking two languages and/or a later age of acquisition of the second language. This may result in weaker and less precise phonological representations of words in memory, which may cause greater retrieval effort during spoken word recognition. To gauge retrieval effort, the present study compared the effects of word frequency, neighborhood density (ND), and level of English experience by testing monolingual English speakers and native Spanish speakers who differed in their age of acquisition of English (early/late). In the experimental paradigm, participants heard English words and matched them to one of four pictures while the pupil size, an indication of cognitive effort, was recorded. Overall, both frequency and ND effects could be observed in the pupil response, indicating that lower frequency and higher ND were associated with greater retrieval effort. Bilingual speakers showed an overall delayed pupil response and a larger ND effect compared to the monolingual speakers. The frequency effect was the same in early bilinguals and monolinguals but was larger in late bilinguals. Within the group of bilingual speakers, higher English proficiency was associated with an earlier pupil response in addition to a smaller frequency and ND effect. These results suggest that greater retrieval effort associated with bilingualism may be a consequence of reduced language experience rather than constitute a categorical bilingual disadvantage. Future avenues for the use of pupillometry in the field of spoken word recognition are discussed.

## Introduction

Spoken word recognition (SWR) is a complex process that requires the encoding of an acoustic signal and subsequent mapping of this information to phonological representations in memory (McQueen, 2007). The ease with which a word can be retrieved from memory depends on the goodness of fit between the signal and the stored representation (which is contingent on the quality of the signal and the quality of the representations; Rönnberg et al., 2013), the memory strength of a word (e.g., Monsell, 1991), and the number of words that partially match the speech signal and, as a result, compete for selection with the target word (Luce and Pisoni, 1998; for a brief review see Weber and Scharenborg, 2012). While this process is effortless under optimal circumstances for monolingual speakers, it may be more challenging for second language (L2) and bilingual speakers. Because bilinguals are exposed to each of their languages less often compared to someone who only speaks one language, this reduced exposure may exert a subtle influence on the recognition process. The present study investigated the influence of memory strength (operationalized here as lexical corpus frequency) and the number of competing words matching the speech signal (operationalized as neighborhood density) on SWR and how these factors interact with language experience (operationalized as language status (monolingual, early and late bilingual) and language proficiency). To this end, the pupil response, a measure of cognitive effort (for reviews see Beatty and Lucero-Wagoner, 2000; Goldinger and Papesh, 2012; Laeng et al., 2012), was recorded while participants matched spoken words to visually presented pictures (i.e., the visual-world paradigm; Tanenhaus et al., 1995).

The pupillary response is interesting to psychologists because of its tight link to the locus coeruleus norepinephrine system (LC-NE; Aston-Jones and Cohen, 2005; Laeng et al., 2012). LC activity has been linked to different cognitive processes such as attention allocation and memory consolidation and retrieval (Sara, 2009; Sara and Bouret, 2012). In psychological research, the pupil response, an indirect index of LC activity (Aston-Jones and Cohen, 2005, p. 421), is often used to measure cognitive effort, or processing load, associated with a task. In a seminal study, Kahneman and Beatty (1966) had participants hold digit strings of varying size in memory. The authors found that the pupil dilated as a function of set size and gradually contracted when subjects were asked to recall the memorized digits. Since then pupillometry has been used to investigate various cognitive processes (e.g., Beatty, 1982; Ben-Nun, 1986; Just and Carpenter, 1993; Võ et al., 2008; Wierda et al., 2012).

As mentioned above, one variable influencing SWR is lexical frequency, viewed by many as the most important determinant of lexical retrieval times (e.g., Murray and Forster, 2004). Frequency effects (FEs) have been found in all domains related to lexical access such as lexical decision, reading, picture naming, and SWR tasks. The effects are often explained in terms of memory strength in that repeated exposure to a word strengthens its lexical representation, which in turn reduces subsequent retrieval times (e.g., Monsell, 1991). FEs have gained attention in the literature on bilingual lexical access, as they may be responsible for the often-reported bilingual disadvantage on verbal tasks. (Early) bilingual speakers are often found to have lower vocabulary knowledge even in their dominant language compared to monolingual speakers (Portocarrero et al., 2007; Bialystok et al., 2009; Bialystok and Luk, 2012). This finding is explained by the fact that bilingual speakers are, on average, exposed less frequently to each of their languages compared to monolingual speakers of either language. This reduced exposure may also be responsible for why bilingual speakers often show longer response latencies compared to monolinguals on tasks such as picture naming (e.g., Gollan et al., 2008; Ivanova and Costa, 2008) and visual word recognition (e.g., Duyck et al., 2008; Lemhöfer et al., 2008; Gollan et al., 2011). It should be pointed out that the bilingual disadvantage in lexical access is typically largest when participants are tested in a late-acquired, non-dominant language (e.g., Duyck et al., 2008; Gollan et al., 2011) but is also present in early bilinguals tested in their first and dominant language (Ivanova and Costa, 2008). These studies generally show that bilingual speakers exhibit a larger FE compared to monolingual speakers, that is, when regressing lexical frequency on response latencies, the slope is steeper for bilinguals. Given that bilinguals are, on average, exposed less often to each of their languages, all words in their mental lexicon will be of lower subjective frequency. And given the logarithmic relationship between lexical frequency and retrieval times (small changes in frequency at the low end of the frequency scale impact lexical access time more than changes at the high end of the scale; Murray and Forster, 2004), reduced exposure will affect recognition of low frequency words more than recognition of high frequency words. This view is expressed in the weaker links hypothesis (Gollan et al., 2008), the frequency-lag hypothesis (Gollan et al., 2011), and the lexical entrenchment account (Diependaele et al., 2013). In addition, Diependaele et al. (2013) hypothesized that vocabulary size would be an indication of memory strength, or lexical entrenchment, of words in the mental lexicon. According to this account, a larger lexicon is associated with generally more entrenched lexical representations. Therefore, individuals with a larger lexicon are expected to have stronger lexical representations compared to individuals with smaller lexicons, especially in the low frequency range. The authors tested this prediction by analyzing response time data from a word identification task (the progressive demasking paradigm) from native (L1) and L2 English speakers. Diependaele et al. found an interaction between frequency and vocabulary knowledge for L1 and L2 speakers. Importantly, the coefficients of this interaction were very similar when native and nonnative participants were analyzed separately, showing that the differences between the groups were continuous rather than categorical. The authors concluded from this study that L1-L2 differences in lexical retrieval could be largely attributed to weaker lexical representations of L2 as a result of reduced L2 exposure (rather than cross-language competition). Further confirming this view is a reading study by Whitford and Titone (2012) who found that more L2 exposure was not only associated with a smaller L2 FE but also a larger L1 FE.

A few studies have investigated FEs by measuring the pupil response during lexical retrieval. Kuchinke et al. (2007) used a lexical decision task while manipulating emotional valence and word frequency. In this study, low frequency words were associated with a larger peak pupil dilation compared to high frequency words. The authors attributed this finding to higher resource consumption for the retrieval of low frequency words. For the domain of language production, Papesh and Goldinger (2012) found that the pupil diameter increased less when naming high frequency words compared to low frequency words. In line with these findings, van Rijn et al. (2012) found that the pupil dilation varied as a function of memory strength. In this study, participants learned paired associates once and were then tested on each pair four times while receiving feedback on their response. The authors found that the pupillary response decreased as a function of repetition and interpreted this finding as showing reduced retrieval effort for stronger memories. Thus the pupil response during lexical retrieval can serve as an index of retrieval effort, reflecting memory strength. One study, however, did not find a reliable FE in the pupil response. Papesh et al. (2012) used a recognition memory paradigm in which participants first heard words and non-words that they were asked to remember and later they were presented with old and new items and had to judge whether an item was in the studied list. The pupil response during the study phase did not differ as a function of frequency but was larger for non-words than words. During the recognition phase, old low frequency words elicited a slightly larger pupil response than old high frequency words. While the main effect of word type was significant, the difference between high and low frequency words was small^1^. This suggests that FEs may not always be found in the pupil response depending on task demands.

Bilingual SWR may not only be slower because words in the bilingual lexicon are of lower subjective frequency but also because of increased competition from similar sounding words. Effects of neighborhood density (ND; the number of words that can be formed by adding, deleting, or substituting one phoneme) is well attested in the monolingual literature on SWR (e.g., Goldinger et al., 1989; Cluff and Luce, 1990; Luce and Pisoni, 1998; Vitevitch and Luce, 1998). A common finding is that words from dense neighborhoods are recognized more slowly and less accurately than words from sparse neighborhoods. To explain this finding, current models of SWR assume that similar sounding words receive activation from the speech signal and compete for selection (McClelland and Elman, 1986; Norris, 1994; Luce and Pisoni, 1998; Norris and McQueen, 2008). Thus more perceptual input is needed for the system to decide between the active candidate words. In the literature on bilingual SWR, research suggests that neighborhood effects are larger in a listener's second language compared to their first language (Bradlow and Pisoni, 1999; Imai et al., 2005). This may be because of reduced sensitivity to phonetic detail (Bradlow and Pisoni, 1999): If words sound more similar to the listener, more words will compete for selection, which will result in longer retrieval times (also see Weber and Cutler, 2004; Broersma and Cutler, 2011). Additionally, bilinguals may have less precise phonological representations of words in long-term memory (Imai et al., 2005) and so the matching of the speech signal to memory representations may be less efficient and result in more retrieval failures. Imai et al. divided their bilingual participants into two groups according to their proficiency in English. They found that the high proficiency group recognized more words from dense neighborhoods than the low proficiency group. Therefore it seems that the effect of ND was attenuated by language proficiency. This may indicate that phonological representations become more precise with greater language experience, resulting in more efficient processing. The manipulation of ND allowed the testing of two hypotheses. Because similar sounding words are assumed to compete for selection, word recognition is harder for words from dense neighborhoods than for words from sparse neighborhoods. Thus, if the pupil response reflects retrieval effort as a result of lexical competition, it should show an effect of ND. Furthermore, if bilinguals experience more competition between similar sounding words, neighborhood effects will be larger for them compared to monolinguals.

To investigate the effects of language experience (i.e., bilingualism), lexical frequency, and ND during SWR, three groups of participants were tested: monolingual English speakers, and early and late Spanish-English bilinguals (see the next section for a detailed description of the participants). In addition, language proficiency was tested as a continuous variable with a standardized test. All bilingual participants learned Spanish as their first language but learned English either early or later in life. English language proficiency was therefore used as a proxy variable for exposure to English over a lifetime as the latter variable is difficult to measure directly. The positive relationship between these two variables has been well established in numerous large scale studies (e.g., Johnson and Newport, 1989; Flege et al., 1999) as well as more controlled studies with bilingual children (Thordardottir, 2011; Hurtado et al., 2013). It was therefore hypothesized that if FEs and ND effects are related to language exposure, they will also be related to language proficiency. Thus the primary research questions were whether the pupil response would vary as a function of language experience, frequency, and ND and whether the size of the FE and the ND effect would interact with language experience.

## Materials and methods

### Participants

Fifty-three participants participated in this study. These participants came from three different groups, English monolingual, early Spanish-English bilingual, and late Spanish-English bilingual. Monolingual was defined in this study as someone who grew up monolingual in an English-speaking environment. Some monolingual participants had taken high school or college language classes and were technically bilingual. However, only three participants in the monolingual group reported fluency in a second language. Although learning a second language may have an influence on one's first language, this influence was considered to be minimal because of the late and infrequent exposure to the second language for those who had learned one. All bilingual participants grew up speaking Spanish but differed in their age at which they started to acquire English. Early bilinguals were born in the USA or arrived before the age of 8. They had received all or most of their schooling in English and had no perceivable accent. Late bilinguals arrived at the age of 18 or later and came from Colombia, the Dominican Republic, Guatemala, Mexico, and Puerto Rico. They had started to learn English in their home countries and had reached levels of English proficiency that allowed them to either study or work at the university (see Table 1 for a description of the participants by group). It should be noted that some of the participants in this group attended English immersion programs in their home countries and had reached high levels of fluency in English. Therefore, the terms early and late bilingual refer more to the environment a participant grew up in (predominantly English or predominantly Spanish). All participants reported normal or corrected to normal vision and normal hearing. Participants were recruited from Michigan State University and received a monetary compensation for their participation. The study protocol was approved by the local institutional review board and participants gave informed written consent.

**Table 1:** **Participant information**.

	**Early bilinguals**		**Late bilinguals**		**Monolinguals**	
	***n* = 17^1^ (8 males)**		***n* = 15 (9 males)**		***n* = 21 (9 males)**	
	**Mean**	***SD***	**Mean**	***SD***	**Mean**	***SD***
Age	21.6^a^	4.9	24.1^a^	7.2	21.9^a^	3.3
Age of arrival in US	1.1^a^	2.4	22.3^b^	5.6	0.1^a^	0.4
Years of formal education	15.0^a^	3.5	15.9^a^	4.2	15.5^a^	1.9
Started learning English	3.5^a^	2.4	6.7^b^	4.6	0.0^c^	0.0
Started learning Spanish	0.2^a^	0.4	0.2^a^	0.4	–	–
English exposure (%)^2^	69.9^a^	16.4	57.3^b^	27.0	96.9^c^	5.9
Years in English environment	19.0^a^	5.6	3.2^b^	3.6	21.9^c^	3.3
Picture vocabulary—English^3^	93.6^a^	10.7	78.5^b^	9.2	99.5^c^	6.9
Verbal analogies—English^3^	104.8^ab^	9.9	99.1^a^	11.1	108.8^b^	6.6
Oral language ability—English^4^	98.8^a^	11.2	84.7^b^	11.1	104.5^a^	7.0
Oral language ability—Spanish^4^	83.0^a^	9.4	99.3^b^	7.5	–	–

Different superscripts indicate significant differences between groups at the p < 0.05 level (determined through robust regression). Same superscripts indicate that differences between groups were not significantly different (p > 0.5).

1 One additional early bilingual speaker was tested but later excluded (see text).

2 Current average exposure to English.

3 Measured with the Woodcock-Muñoz Language Survey-Revised, a standardized test with a population mean of 100 and a SD of 15.

4 Composite score of picture vocabulary and verbal analogies.

### Testing materials

#### Language proficiency

Language proficiency was assessed using two subtests of the Woodcock-Muñoz Language Survey—Revised (Woodcock et al., 2005), picture vocabulary and verbal analogies. In the picture vocabulary test, participants are asked to name pictures of objects and in the verbal analogies test, participants are asked to complete analogies of the form A is to B as C is to … The test provides age-normed standard scores for each test in addition to a composite score, oral language ability, which reflects broad language ability^2^. Both bilingual groups also completed the tests in Spanish. Results from a listening test that bilingual participants also completed are not reported here because the monolingual participants did not complete this part. In addition to the language proficiency test, participants completed a language background questionnaire, which was taken from Marian et al. (2007).

#### Stimuli

Pictures for the eye-tracking experiment came from Cycowicz et al. (1997; see Table A1 for a list of all stimuli and their lexical characteristics). Information about word frequency was taken from Brysbaert and New (2009) and was used as a continuous variable. Two stimuli (*can* and *well*) were later dropped from the analysis because no reliable frequency estimates could be found for the noun frequencies. Information about the number of phonological neighbors was taken from the English lexicon project (Balota et al., 2007). A female speaker of American English spoke all picture names in isolation, which were recorded in a soundproof booth over a single channel. Sound stimuli were then normalized in Praat.

**Figure 1:**
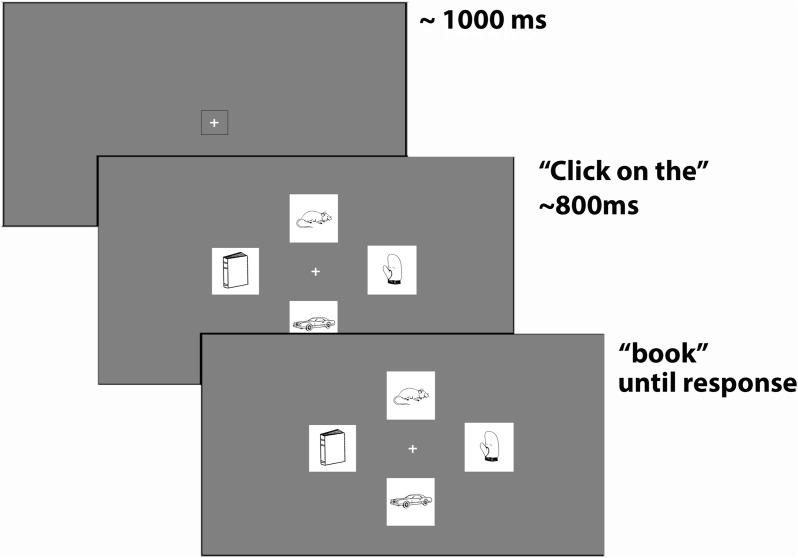
**Trial procedure**. A trial started with a fixation cross. A box around the fixation cross turned red when a fixation was detected. Four pictures appeared while participants heard “Click on the [target word].” Pictures had been on the screen for about 800 ms at the onset of the target word. A trial ended when a mouse response was detected.

As is common in visual-world paradigm studies (e.g., Allopenna et al., 1998), target pictures appeared with three distractor pictures (see Figure 1). For all trials, care was taken that the three distractor pictures did not overlap with the target in shape or meaning. The original visual-world paradigm experiment also included trials (*k* = 27) for which the target appeared with a Spanish phonological cohort competitor [PC; e.g., target: envelope – PC: enchufe (plug)]. This manipulation was not of interest for the present analysis but those trials were included here to achieve greater power to find effects. In a different condition, targets appeared with an English PC but this manipulation had an effect on the pupil response (see Footnote 3 in the Results section), and so these trials (*k* = 14) were not included in the analysis. All trials with a PC were repeated once with a control picture (no phonological overlap) and these trials were also included in the analysis. Another 35 trials were not paired with a PC and only appeared once. This resulted in a total of 76 unique stimuli of which 41 were repeated for a total of 117 experimental stimuli, 103 of which were entered into the final analysis.

### Apparatus

Pupil size was recorded with a Tobii TX300 eye tracker, sampling at 300 Hz from both eyes, and stimuli were presented on a 23”, 1920 × 1080 pixel widescreen monitor. The pupil diameter output of the TX300 is corrected for the spherical corneal magnification effect and distance to the eye (Tobii TX 300 product brochure). Stimuli were presented in E-Prime 2.0 (Psychology Software Tools, Sharpsburg, PA) using the E-Prime extension for Tobii.

### Procedure

The tests reported here were part of a larger study that investigated bilingual lexical access. Participants completed the following tasks and tests in this order: consent form, language background questionnaire, verbal fluency test, WMLS-English, picture naming^3^, eye tracking (visual-world paradigm), WASI, numerical Stroop, and WMLS-Spanish (bilinguals only; the tests not reported here were part of a separate study). For the eye-tracking experiment, participants were seated in a dimly lit room at approximately 60 cm away from the eye tracker. Stimuli were played back to participants binaurally via headphones (Audio-Technica ATH-M50). A standard five-point calibration of the eyes was performed at the beginning of the experiment. Each trial started with a fixation cross that participants were asked to fixate for 1 s. A box around the fixation cross turned red when a fixation was detected to ensure that participants' eyes were within the field of the eye tracker. Then four pictures, each 6.1 × 5.7 cm large (subtending 5.8 × 5.4° at a viewing distance of 60 cm), appeared together and participants heard “Click on the [target picture].” The duration of the carrier sentence was 688 ms and the target pictures were on the screen for approximately 800 ms at the onset of the target word. Participants saw a total of 122 trials but the first five trials of each participant constituted test trials and were discarded for the analysis. A trial ended when the participant made a mouse response by clicking on a picture (see Figure 1). Trial order was randomized for each participant. In addition, the position of the four pictures was randomized across trials and participants so that the position of the target picture was not predictable. This procedure also ensured that any effects associated with target words were not confounded by picture position. Targets that had a PC were repeated so that they appeared once paired with a competitor picture and once without whereby the competitor picture (e.g., mountain) was replaced with a phonologically unrelated picture, which was the competitor for a different target. This procedure is common in visual-world paradigm studies and ensures that the only variable that differs between conditions is competitor present or absent. Conditions with PC were counterbalanced so that half of the targets appeared with an unrelated picture first and then with a PC whereas the other half appeared with a PC first. Block order was counterbalanced across participants.

### Data reduction, cleaning, and selection

Because of the large amount of data resulting from the eye tracker output, data were down sampled. To this end, the pupil diameters from 4 consecutive samples were binned and averaged, resulting in a temporal resolution of about 13.33 ms. Bins containing observations with low validity (coded by the Tobii software) were coded as missing values as were observations where the change in pupil diameter from one bin to the next exceeded 0.1 mm. This was done separately for the left and right eye. Missing values were then replaced by linear interpolation. After this process, data were smoothed with a five-point weighted moving-average smoothing function.

The dependent variables used in the present study were the peak amplitude (PA), and peak latency (PL), which were calculated for each trial (programmed in Python) as is common in studies investigating the pupil response (e.g., Zekveld et al., 2010). The PA refers to the largest dilation in a trial and PL is the time elapsed from word onset to the PA. In addition, a baseline diameter was calculated by averaging over the first 100 ms before the onset of the target word. This baseline measure was then subtracted from the PA to account for differences in pupil diameter at the onset of a trial.

Observations from both eyes correlated highly for PL (*r* = 0.87), PA (*r* = 0.92), and invalid observations (*r* = 0.95). To reduce the noise inherent in each measure, measurements from both eyes were averaged. Trials with response times 3 SDs above the mean (>3 s) were excluded (1.9%). Then trials with more than 30% missing observations (3%), trials for which the baseline amplitude was higher than the PA (5%), and inaccurate trials (2.3%) were excluded. After these exclusion criteria were applied, subjects had, on average, 86% valid trials (*SD* = 9, range = 97–60). Data from one subject were excluded after a visual inspection of the data. The average pupil diameter of this participant decreased after target word onset while all other participants showed the opposite pattern. This resulted in very short PLs (around 266 ms), which are unlikely to reflect processes associated with SWR but suggest measurement error. Leaving this participant in did not change the pattern of results.

### Analysis

Statistical analyses were performed in the statistics program *R* (R Core Team, 2013) using the lme4 package (Bates et al., 2013). Models were fit with random intercepts for subjects and items and random slopes for the FE for both items and subjects except in cases where such a model did not converge or intercepts and slopes were perfectly correlated (see Baayen et al., 2008). Because the effect of interest may be confounded by other variables, several control variables were added to the model. These were the number of phonemes of a word, whether a target picture had been previously named, and whether a target picture was repeated (see Footnote 2). In addition, some target words were cognates of their Spanish translation equivalent and so cognate status was also entered as a control variable.

## Results

**Figure 2:**
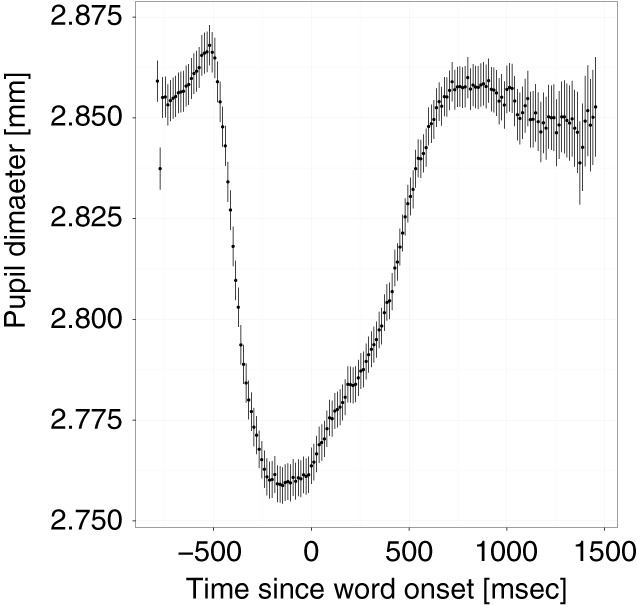
**Grand average of the pupil diameter over the course of a trial**. Zero marks the onset of the target word. Vertical lines around means show the standard error for each observation.

Figure 2 shows the pupil diameter averaged across participants and trials. The figure shows a contraction of the pupil occurring at about −500 ms followed by a relatively flat curve and an increase in pupil diameter at the onset of the target word. The initial dip is likely in response to the change in luminance created by the appearance of the pictures (see Figure 1). However, the graph suggests that participants' eyes had adapted to the new luminance level by the time they heard the target word. The mean trial length was 1204 ms (*SD* = 389) and the mean PA occurred on average at 867 ms (*SD* = 428) after target word onset with an average dilation of 2.95 mm (*SD* = 0.38; baseline corrected mean = 0.20 mm, *SD* = 0.15). Note that these values do not correspond to those in Figure 2 because the peaks occurred at different times for different trials. The results of the statistical analyses will be reported for PL first and then for PA^4^.

### Peak latency

**Figure 3:**
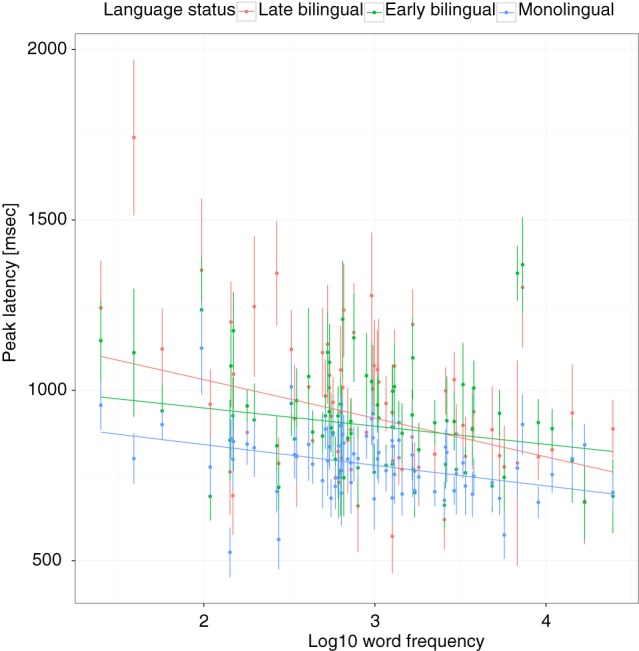
**Peak latencies as a function of lexical frequency and language status**. Vertical lines and dots show the mean and standard error of individual items. Regression lines show the best fit for each group.

**Figure 4:**
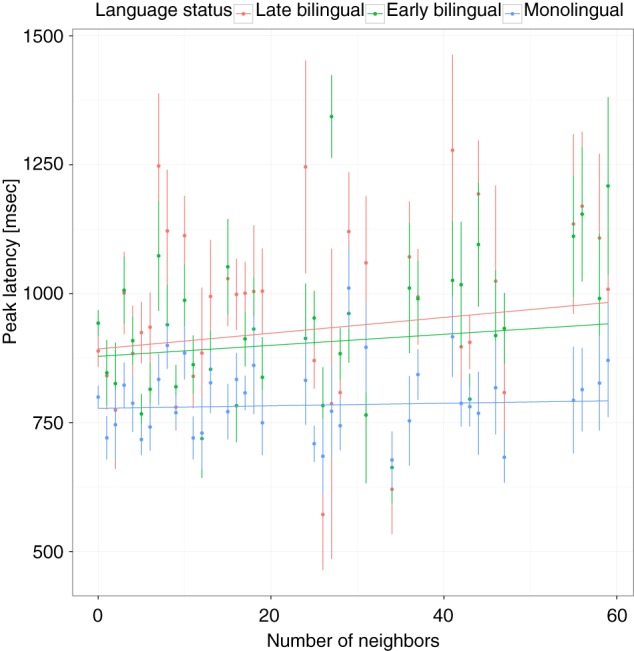
**Peak latencies as a function of neighborhood density**. Vertical lines and dots show the mean and standard error of individual items. Regression lines show the best fit for each group.

For the analysis, a regression model was built by entering all predictor and control variables. The results are shown in Table 2. The main effect of language status (monolingual, early bilingual, late bilingual) was significant. Compared to the late bilinguals, the PLs of monolinguals occurred, on average, 156 ms earlier, but early bilinguals were not significantly different from late bilinguals. Using early bilinguals as the reference category showed that the difference between monolinguals and early bilinguals was also significant (*b* = −124, *SE* = 41, *p* < 0.0036). The interaction between language status and frequency showed that late bilinguals had a FE of −84 ms for an increase of 1 SD in frequency and this effect was attenuated by 47 ms for early bilinguals and 52 ms for monolinguals. When early bilinguals were used as the reference category, it showed that the difference between monolinguals and early bilinguals was not significant, *b* = 5, *SE* = 14, *p* = 0.7244, suggesting that the FE in these two groups was the same (see Figure 3). The interaction between language status and ND showed an effect of 66 ms for 1 SD increase in ND for the late bilinguals. Compared to this group, the effect for early bilinguals was not significantly different but the ND effect was attenuated in monolinguals by −36 ms. When the group of early bilinguals was used as the reference category, the effect of 1 SD increase in ND was 58 ms (*SE* = 15, *p* = 0.0001), which was not significantly different from late bilinguals, *b* = 14, *SE* = 16, *p* = 0.3711, or monolinguals, *b* = −22, *SE* = 14, *p* = 0.1111 (see Figure 4).

**Table 2:**
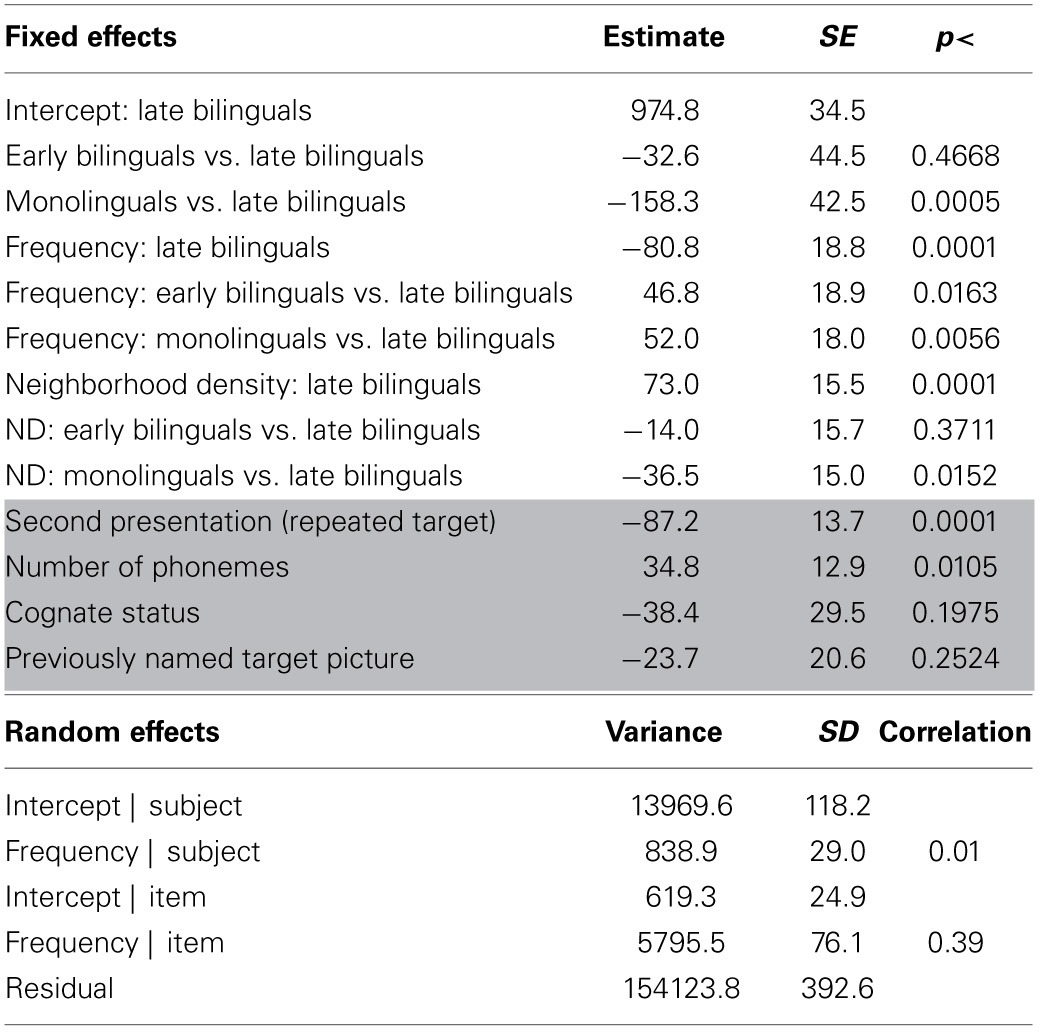
**Results for the analysis of peak dilation latencies**.

p-values were calculated using the lmerTest package (Kuznetsova et al., 2013). Control variables are shown in gray. All continuous variables were transformed into z-scores so that the estimate of the predictor variable shows the change associated with an increase of 1 SD. Second presentation: some items were repeated and the estimate shows the reduction in latencies for the repeated item. Cognate status: whether a target was a cognate of its Spanish equivalent. Previously named target picture: some pictures also appeared in a picture-naming task right before the eye-tracking experiment. The estimate shows the change for an item that was previously named compared to an unnamed item.

**Figure 5:**
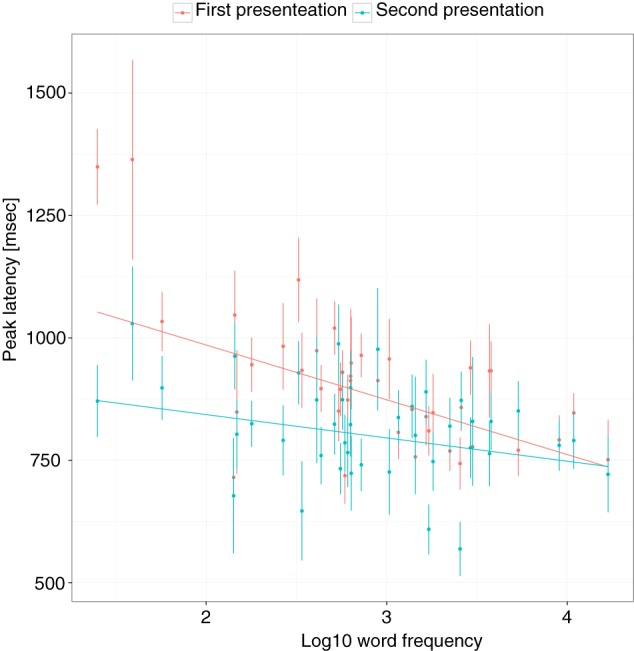
**The frequency effect as a function of target repetition**. The effect is shown for repeated words only. Vertical lines and dots show the mean and standard error of individual items. Regression lines show the best fit.

Because some targets were repeated, the effect of repetition was further investigated. When only unrepeated trials (i.e., only the first presentation of trials that had not been previously named) were included (*k* = 40), the main effect of language status and the interaction with frequency remained significant. Results indicated that the PL for late bilinguals occurred at 1023 ms (*SE* = 40). The PL of early bilinguals was not significantly different, *b* = −49, *SE* = 50, *p* = 0.3357, but the PL of monolinguals was significantly faster, *b* = −195, *SE* = 48, *p* = 0.0002. In late bilinguals, 1 SD increase in frequency was associated with an earlier peak, *b* = −111, *SE* = 27, *p* < 0.0001, and this effect was reduced in early bilinguals by 62 ms (*SE* = 27, *p* = 0.0231) and by 58 ms (*SE* = 26, *p* = 0.0264) in monolinguals. The difference between early bilinguals and monolinguals was again not significant, *b* = −4, *SE* = 24, *p* = 0.8399. From this analysis it appears that FEs were larger for unrepeated trials compared to the full data set. To investigate this further, only those targets that were repeated were analyzed. The main effect of frequency, *b* = −91, *SE* = 14, *p* < 0.0001, and repetition, *b* = −85, *SE* = 14, *p* < 0.0001, were significant. In addition, the interaction between frequency and repetition was significant, *b* = 51, *SE* = 14, *p* = 0.0003, indicating that the facilitatory effect of repetition was largest for low-frequency words (see Figure 5). The effect of ND was no longer significant in the data set with only unrepeated trials, *b* = 30, *SE* = 28, *p* = 0.2872, or only repeated trials, *b* = 21, *SE* = 17, *p* = 0.1938. Note that the sign of the effect was still in the predicted direction but there may not have been enough power to find a reliable effect due to the lower number of trials in these analyses. There was no interaction between ND and repetition in either the full or the reduced data set (*ps* > 0.5).

**Figure 6:**
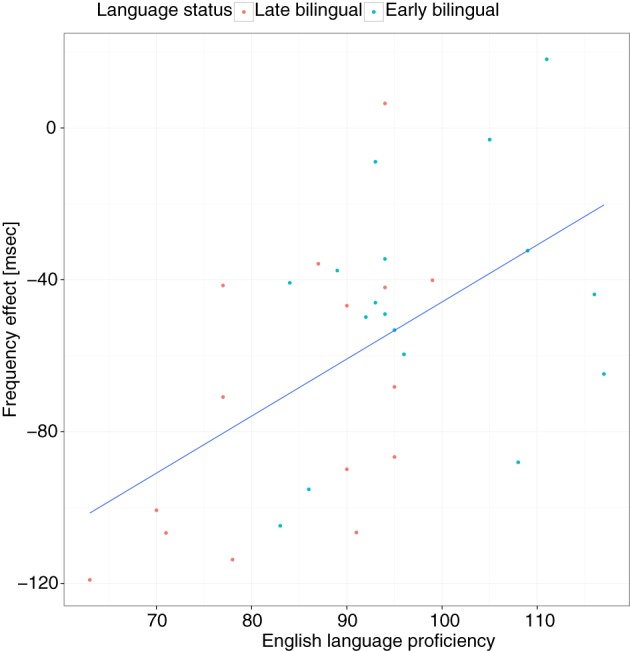
**Frequency effects as a function of language proficiency and language status**. The y-axis shows the frequency effect for 1 SD change in log10 lexical frequency, extracted from the mixed-effect regression model run on the raw data of the bilingual participants (see text). Each dot represents one participant. The regression line shows the best fit.

**Figure 7:**
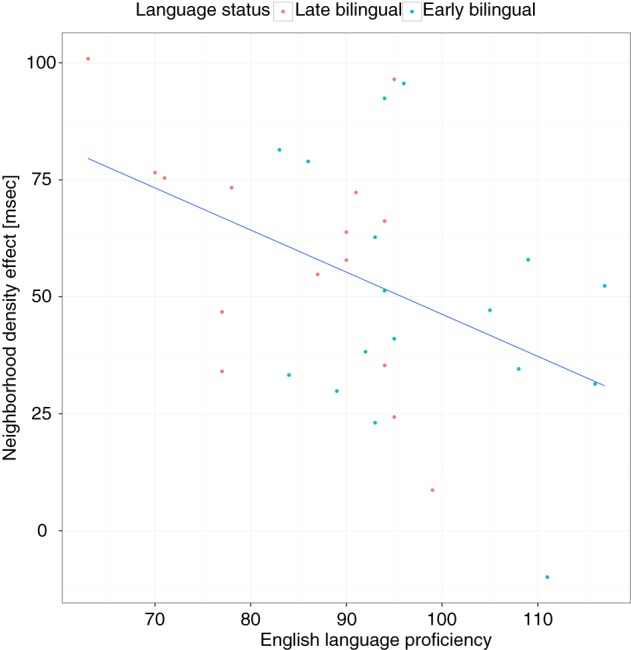
**Neighborhood density effects as a function of language proficiency and language status**. The y-axis shows the neighborhood density effect for 1 SD change in neighborhood density, extracted from the mixed-effect regression model run on the raw data of the bilingual participants (see text). Each dot represents one participant. The regression line shows the best fit.

The previous analyses indicated that frequency and ND effects were modulated by language status. To investigate the hypothesis that language experience attenuates these effects, follow-up analyses were conducted with the bilingual groups only and English proficiency was used as a continuous variable rather than language status. In this model, the interaction between English proficiency and frequency and proficiency and ND were significant (see Table 3). This indicates that higher proficiency was associated with smaller frequency and ND effects. These interactions can be further illustrated by running a model in which the effects for frequency and ND are allowed to vary by subject (i.e., a random slopes, random intercepts model). These slope adjustments then show the effect size for each participant. The correlation between the FE and English proficiency was significant, *r*_(30)_ = 0.53, 95% CI = [0.23, 0.75], *p* = 0.0015 (see Figure 6), as was the correlation between the ND effect and proficiency, *r*_(30)_ = −0.45, 95% CI = [−0.69, −0.12], *p* = 0.0093 (see Figure 7). When these same analyses were run with the monolingual participants only, neither of these interactions was significant (*ps* > 0.66). However, the main effect of frequency, *b* = −34, *SE* = 10, *p* = 0.0012, and ND, *b* = 32, *SE* = 14, *p* = 0.0248, remained significant.

**Table 3:**
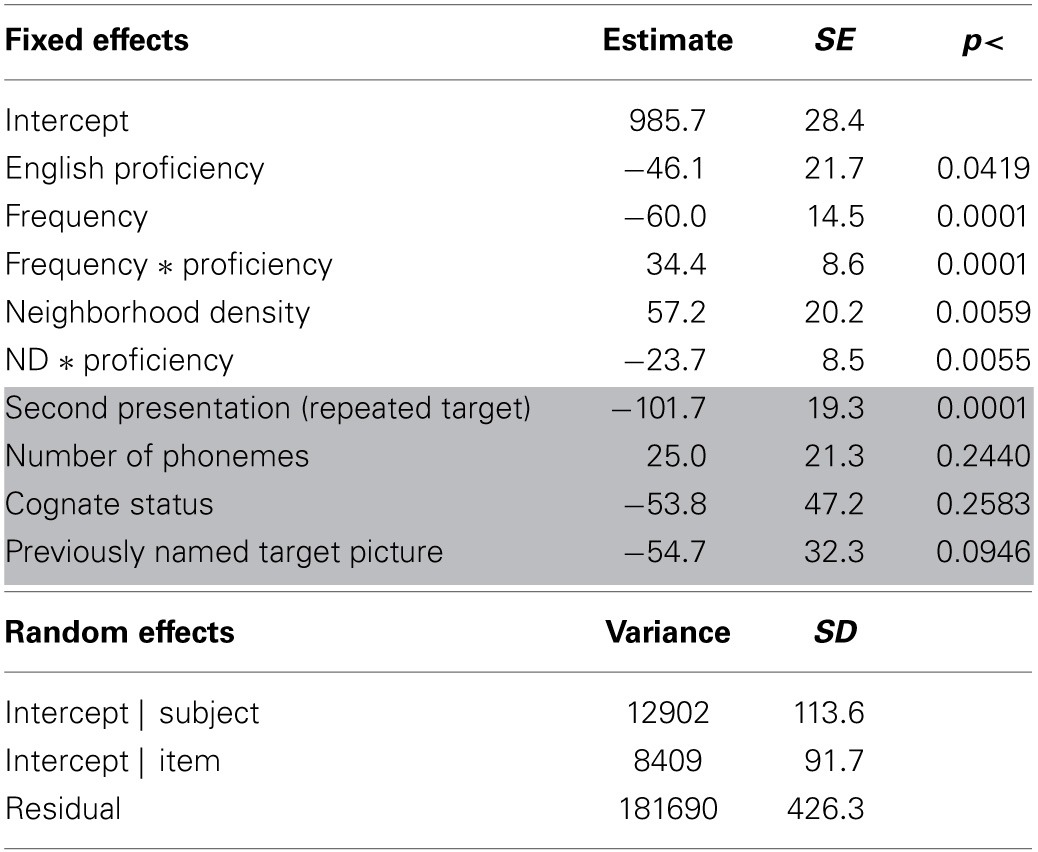
**Results for the analysis of peak dilation latencies—bilingual participants**.

See Table 2 for explanations.

### Peak amplitude

For the analysis of the PA, variables were entered into the model in the same way as in the previous analysis (see Table 4). Language status was not significant, indicating that the mean PAs of each group were not significantly different from each other. The interaction between frequency and language status showed a FE of 0.015 mm for late bilinguals. This effect was reduced by 0.013 and 0.014 mm for early bilinguals and monolinguals, respectively. When monolinguals or early bilinguals were used as the reference category, the FE was not significantly different from zero in either group (*ps* > 0.64). The main effect of ND was significant, indicating that a denser neighborhood was associated with a larger pupil diameter. The interaction between ND and language status was not significant and was therefore dropped from the model.

**Table 4:**
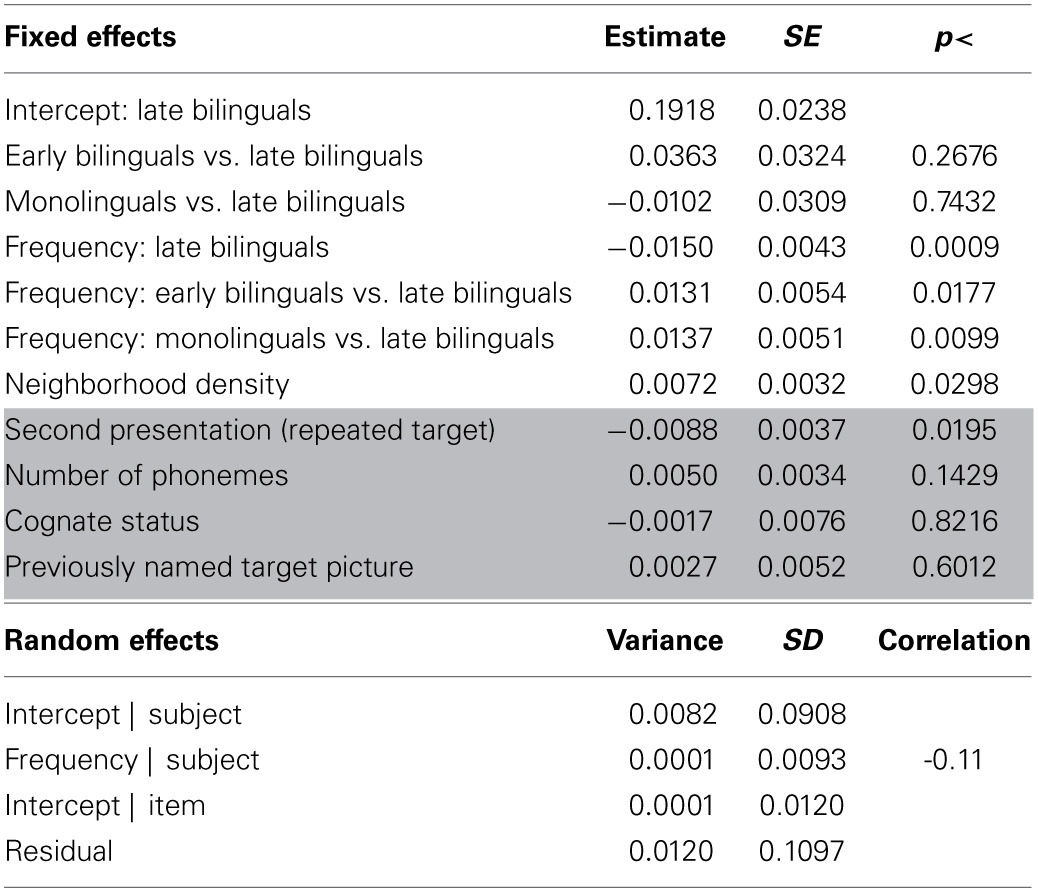
**Results for the analysis of peak dilation amplitudes**.

See Table 2 for explanations.

As in the analysis of PLs, the effect of repetition was further investigated. Using only unrepeated trials, the results showed that only the FE in late bilinguals was significantly different from zero, *b* = −0.018, *SE* = 0.006, *p* = 0.0039. The FE in monolinguals was significantly different from late bilinguals, *b* = −0.013, *SE* = 0.007, *p* = 0.0450, but not from early bilinguals, *b* = −0.003, *SE* = 0.006, *p* = 0.6760. The FE in early bilinguals was not significantly different from late bilinguals, *b* = −0.011, *SE* = 0.007, *p* = 0.1260, showing that the FE of early bilinguals was in between monolinguals and late bilinguals. The effect of ND was not significant in this reduced data set (*p* = 0.946). When considering the effect of repetition by analyzing only those trials that were repeated, the effect of repetition, *b* = −0.010, *SE* = 0.004, *p* = 0.0122, and frequency, *b* = −0.011, *SE* = 0.003, *p* = 0.0010, and their interaction, *b* = 0.009, *SE* = 0.004, *p* = 0.0187, were significant. This again showed that the facilitative effect of repetition was larger for low frequency words compared to high frequency words. The effect of ND remained significant, *b* = 0.006, *SE* = 0.003, *p* = 0.0283, and did not interact with the effect of repetition (*p* = 0.2771).

The previous analysis of the full data set was again followed up with a separate analysis of the bilingual speakers only. The interaction between frequency and English proficiency was significant, indicating that FEs were reduced with increased proficiency (see Table 5). Contrary to the analysis of PLs, the interaction between proficiency and ND was not significant. When a model without these interactions was run and the slope adjustments of the FE for individual subjects were extracted, the correlation between proficiency and the slope estimates was significant, *r*_(30)_ = 0.47, 95% CI = [0.15, 0.71], *p* = 0.0062. When the monolingual group was run separately, the only effect that remained significant was that of repetition, *b* = −0.0124, *SE* = 0.0050, *p* = 0.0136.

**Table 5:**
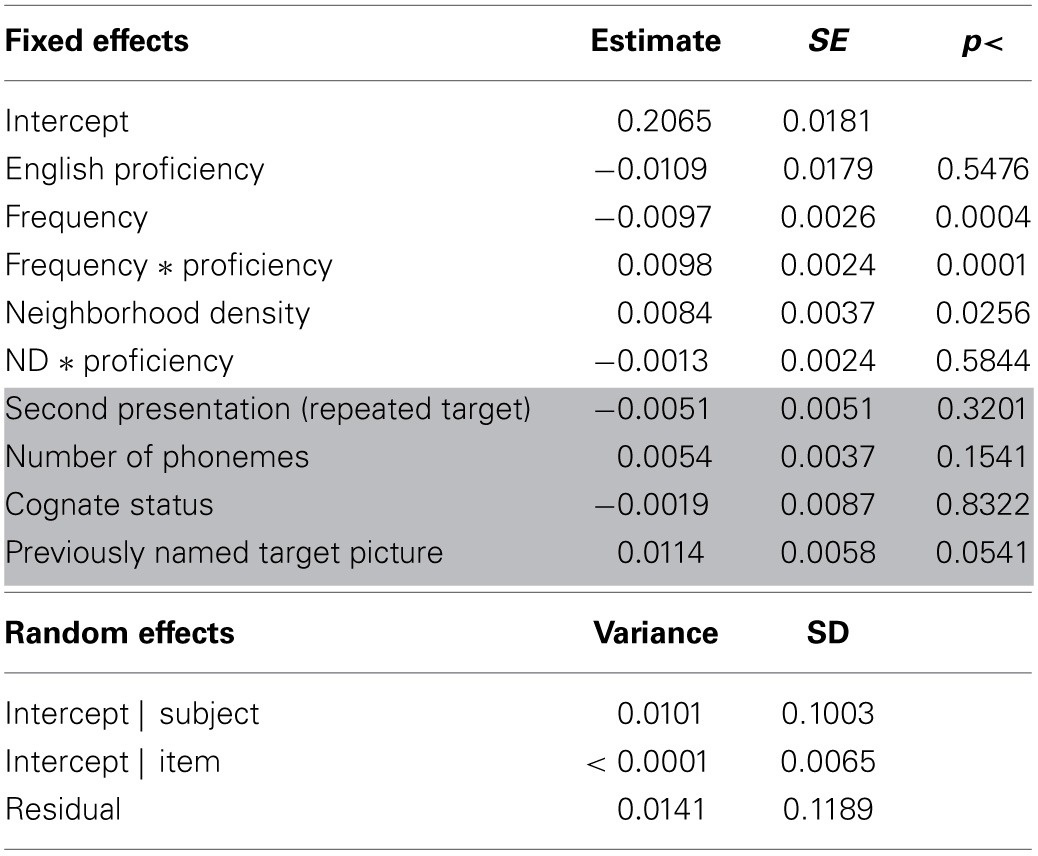
**Results of the analysis of peak dilation amplitudes—bilingual participants**.

See Table 2 for explanations.

## Discussion

### Frequency effects

Results from previous studies investigating FEs suggest that the pupil response during information retrieval is an indication of retrieval effort reflecting the strength of a memory trace (Kuchinke et al., 2007; Papesh and Goldinger, 2012; Papesh et al., 2012; van Rijn et al., 2012). The present study found an association between language proficiency and lexical frequency in a group of bilingual speakers, such that higher English proficiency was associated with a smaller FE. Assuming that language proficiency is closely related to language exposure in bilinguals (Thordardottir, 2011; Hurtado et al., 2013), language proficiency is likely a proxy variable for language experience over the course of a lifetime. Thus the present findings suggest that, in the group of bilingual participants, reduced language experience was associated with weaker connections between phonological and lexical representations. This is in line with previous research on language production and visual-word recognition showing that more use of a language is usually associated with a smaller FE (e.g., Duyck et al., 2008; Gollan et al., 2008, 2011; Ivanova and Costa, 2008; Whitford and Titone, 2012). To the best of my knowledge, the present study is the first to extend these findings to the domain of SWR. And given the relationship between memory strength and the pupil response, the present results may been seen as more direct evidence to explain the bilingual disadvantage in lexical access in terms of weaker links (Gollan et al., 2008) or lexical entrenchment (Diependaele et al., 2013).

The present study, however, also presents some evidence that less frequent exposure to a language may not be the only reason for a bilingual disadvantage in lexical access: The magnitude of the FE in early bilinguals was the same as in monolinguals, while the main effect of language status was significant. While there are currently no studies on bilingual SWR to compare these findings to, they resemble those reported in Gollan et al. (2011 Exp. 2) for lexical decision. When comparing early Spanish-English bilingual to English monolingual speakers, the FE in both groups was not significantly different while the monolinguals tended to be overall faster (this effect was marginally significant; Gollan et al., 2011, p. 196). This is in contrast to many language production studies (e.g., Exp. 1 in Gollan et al., 2011) that usually show a larger FE in early bilingual speakers compared to monolinguals, even when they are tested in their first and dominant language (Ivanova and Costa, 2008). It may be, therefore, that for word recognition, early bilinguals who are tested in the language they are dominant in and exposed to most of the time will show FEs similar to monolinguals.

As in Diependaele et al. (2013), the interaction between proficiency and frequency was significant, indicating that lexical representations in bilinguals may be less entrenched due to reduced language exposure. According to this view, the bilingual disadvantage does not stem from speaking two languages *per se* but from being exposed to each language less frequently. Thus, also monolinguals should show a larger FE as a function of reduced language exposure. Diependaele et al. (2013) found this to be true, the interaction between frequency and proficiency was significant for monolinguals as well. This is in line with previous studies on visual word recognition that found a relationship between word frequency and vocabulary knowledge (Yap et al., 2012) or print exposure (Chateau and Jared, 2000) in monolingual English speakers. It is also in line with Whitford and Titone (2012) who found that more L2 exposure was associated with a larger L1 FE in reading. In the present study, however, the interaction between proficiency and frequency was not significant in monolinguals. This may be because monolingual speakers are more homogeneous concerning the amount of exposure to spoken English but more heterogeneous with regard to print exposure. It may be, however, that testing monolingual participants on a wider range of low frequency words would reveal such an interaction.

**Figure 8:**
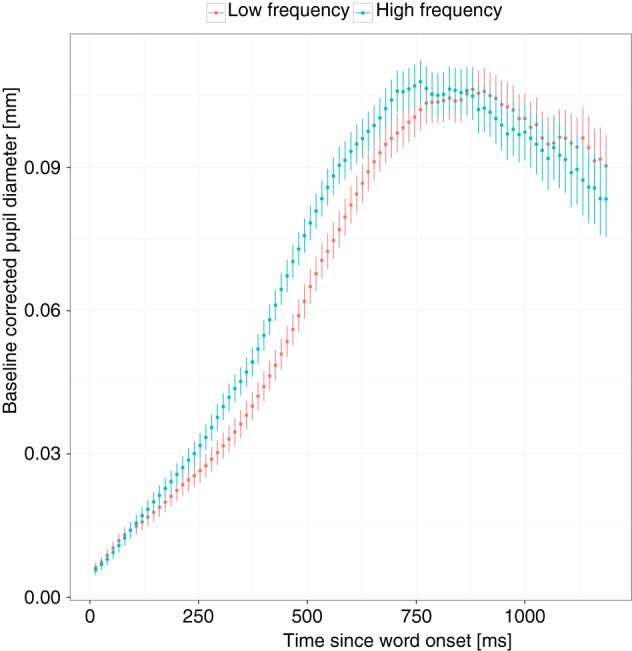
**The frequency effect for monolingual participants**. The x-axis shows the time in milliseconds since word onset and the y-axis the baseline corrected pupil diameter. To illustrate the effect, words were divided into high and low frequency words based on a median split. Vertical lines around means show the standard error for each observation.

The important finding of the present study was the interaction between proficiency and frequency in the bilingual group. This interaction was significant in the analysis of PAs and PLs, showing that higher English proficiency was associated with a smaller FE. However, when looking at the monolingual participants, the FE was only significant in PLs but not PAs. This finding seems to be at odds with Kuchinke et al. (2007) who found a FE in PAs. These differences may be explained by the fact that frequency in this study was used as a dichotomous variable with a large difference between high and low frequency words, whereas frequency was a continuous variable in the present study. The effect size of the pupil response in Kuchinke et al. was rather small (Cohen's *d*, calculated from the means and standard deviations reported in the paper, was between 0.11 and 0.21 in the different conditions) and so the range of frequencies in the present study may have been too small to find the effect. However, when comparing the trajectories of the pupil response of the present study and Kuchinke et al., they look quite different. Figure 8 shows the pupil response to high and low frequency words (based on a median split) for the monolingual participants. A FE appears early on and is characterized by a later peak for low frequency words whereas the amplitude of the peak appears to be the same. In Kuchinke et al. (2007, Figure 1), on the other hand, FEs appear later (at ~600 ms) and are characterized by a lower PA but similar PLs. These differences may be explained by the different tasks used, that is, lexical decision vs. SWR. In Kuchinke et al., FEs may have been associated with “processes of response selection and execution” (p.137), whereas in the present study, FEs appeared before a mouse response was made. In another study (Papesh et al., 2012), participants listened to high and low frequency words (study phase) without giving a response while the pupil size was recorded. These researchers did not find a significant difference in the PA for low and high frequency words. Thus further studies may be needed to determine how an overt vs. no overt response influences the trajectory of FEs when measuring the pupil response.

A further finding of the present study related to frequency was that repetition facilitated the recognition of low frequency words more compared to high frequency words, which was expressed in a repetition by frequency interaction. This interaction was significant for both PAs and PLs and was present in all three groups and suggests that repeated items could be retrieved from memory with less effort. This repetition effect is consistent with behavioral studies (e.g., Scarborough et al., 1977) and research using pupillometry (van Rijn et al., 2012). It is in contrast, though, to the pupil old/new effect reported in Võ et al. (2008). Võ et al. first presented participants with a list of words that they were asked to remember. In a later recognition phase, participants were presented with previously studied and new words. Results showed that the pupil response was larger to old compared to new items. This difference is again likely due to different task demands. Whereas participants in the present study had to recognize the target word and match it to a picture, participants in Võ et al. had to make old/new judgments. Because the pupil response has been associated with different emotional and cognitive states (e.g., Graur and Siegle, 2013), seemingly similar tasks may elicit different pupil responses based on different underlying cognitive processes.

### Neighborhood density effects

Many studies on SWR have shown that words with many neighbors are recognized more slowly compared to words with no or few neighbors (e.g., Luce and Pisoni, 1998). Because of this robust finding, SWR is usually thought of as a competitive process, that is, words that partially match the speech signal receive activation and compete for selection (e.g., Dahan and Magnuson, 2006; McQueen, 2007). The present study contributes to this literature by showing that neighborhood effects in SWR can be observed in the pupil response. Assuming that the pupil response is an indication of retrieval effort, the results show that words from sparse neighborhoods are retrieved with greater ease compared to words from dense neighborhoods. And in line with previous research (Bradlow and Pisoni, 1999; Imai et al., 2005), the present findings suggest that neighborhood effects are modulated by L2 proficiency. An effect of ND on PLs was found that interacted with language proficiency in the bilingual speakers, showing that lower proficiency was associated with slower processing. Thus, the present study extends the results of Bradlow and Pisoni (1999) and Imai et al. (2005) by showing that ND does not only influence recognition accuracy of words presented in noise but also slows down the word recognition process under optimal listening conditions. Concurring with Bradlow and Pisoni (1999), less language experience may result in reduced sensitivity to acoustic phonetic cues. This would result in more similar-sounding words that partially match the speech signal and thus compete for selection (c.f. Broersma and Cutler, 2011; Weber and Broersma, 2012). Or it may be that speakers with less language experience have less precise phonological representations of words in long-term memory, as Imai et al. (2005) suggest. This explanation is also in line with the entrenchment account (Diependaele et al., 2013): Less precise phonological representations in memory will lead to mismatches between the speech signal and the stored representations, which may result in a greater processing cost (also see Rönnberg et al., 2013).

The results reported here suggest that L2 proficiency may be associated with greater competition of similar sounding words and weaker memory representations as a result of reduced language experience. Thus differences between monolingual and less proficient L2 listeners may represent “cumulative effects of lesser efficiency at all levels of processing” (Cutler et al., 2004, p. 3676) from early perceptual processes to retrieving information from memory. This may explain why the monolingual participants in the present study were overall faster (i.e., shorter PLs), even when differences in the frequency and ND effect were controlled for. At the same time, the present results show that the effects of bilingualism are not categorical but are modulated by language experience. A further source of processing differences between monolingual and bilingual speakers may be cross-language activation, that is, not only words in the target language may compete for selection but also words from the irrelevant language. Evidence for cross-language activation during SWR comes from visual-world paradigm studies. In these studies, participants are presented with pictures, with one of the pictures being a cross-language onset (or cohort) competitor of the target word that is heard. These studies show that bilingual listeners initially also tend to look at the cross-language competitor, suggesting that the speech signal activates words in both lexicons (Spivey and Marian, 1999). The effect, however, is not always found and may depend on the proficiency in the irrelevant language (Marian and Spivey, 2003; Weber and Cutler, 2004; Blumenfeld and Marian, 2013; Mercier et al., 2014). It may also depend on the similarity of the sound inventory between languages. Ju and Luce (2004) used the visual-world paradigm with Spanish-English bilingual participants and manipulated the voice onset time (VOT) of target words (English has a longer VOT than Spanish). Participants were tested in Spanish, their first language, but they were highly proficient in English (they appear to be comparable to the early bilingual group in the present study). When the target VOT was Spanish-like, the authors found no evidence for cross-language activation (e.g., when the target was playa (beach), participants did not look at a picture of pliers more than to an unrelated control picture). Only when VOT was English-like did participants also look at the cross-language competitor. Assuming that the bilinguals in the present study with lower English proficiency perceived the English target words less native-like (i.e., English /p/ and Spanish /p/ sound more alike), they may have experienced additional competition from Spanish words. Thus in the present study, the stronger ND effect in less proficient bilingual speakers may be explained by additional cross-language competition. However, as a study by Vitevitch (2011) suggests, there are only few English words that have Spanish neighbors (~4%) and the mean increase in ND when Spanish neighbors were considered was only 1.55, a negligible effect. Therefore the effect of cross-language competition, if present, was likely not large. Based on this study, Vitevitch also reasoned that it may be unnecessary to assume an additional inhibition mechanism to prevent cross-language interference (c.f. Green, 1998) because the number of words competing for selection will only be slightly larger in bilinguals (Vitevitch, 2011, p. 170). However, the present study does not provide evidence for or against cross-language interference or inhibition of the irrelevant language and so it should be acknowledged these factors may also have influenced the present results.

### Limitations and future research

With regard to the present findings pertaining to a bilingual disadvantage, it should be pointed out that English was the second learned language for all participants, even though the early bilinguals were exposed to English from an early age on and later became dominant in that language. Thus these bilinguals are comparable to those tested in studies by Gollan et al. (2005, 2008, 2011) but differ from bilinguals growing up in bilingual regions such as Catalonia or Quebec. The latter often stay dominant in their first acquired language while attaining high levels of proficiency in their L2. However, previous studies suggest that large amounts of L2 exposure also influence L1 processing in bilinguals who stayed dominant in their first acquired language (Ivanova and Costa, 2008; Whitford and Titone, 2012). Thus the present results may be applicable to a wide range of bilinguals. In such a population, however, the relationship between L1 proficiency and L1 processing may not be as strong as in the present study because such bilinguals will likely be more homogeneous with regard to their L1 proficiency. Rather, it may be the amount of L2 exposure over a lifetime that influences L1 processing in those bilinguals as the results from Whitford and Titone (2012) suggest. But given the relatively small sample size in the present study and the novelty of the pupil response as a dependent measure in SWR, more research is needed before more far-reaching conclusions can be drawn.

One limitation of the present study with regard to the analysis of PA was that no upper and lower baseline measures of participants' pupil diameters in darkness and maximum illumination were taken (see, e.g., Zekveld et al., 2010). Such minimum and maximum values of the pupil diameter for each participant can be used to normalize the pupil response to better account for individual differences. Another potential limitation of the study is that the appearance of the pictures created a change in luminance (see Figure 1). Although there was an interval of 800 ms between the appearance of the pictures and the onset of the target word that allowed participants' eyes to adjust, future studies combining the visual world-paradigm with pupillometry should avoid any changes in brightness. Despite these limitations, the present study has shown that pupillometry can be used to investigate SWR in monolingual and bilingual populations.

Assuming that the pupil response reflects word retrieval processes and may therefore be seen as an indication of retrieval effort, pupillometry may offer new insights to language researchers. Thus the present results may be seen as more direct evidence for the hypothesis that bilinguals have weaker connection strengths between semantics and phonological representations than reaction time measures because of the close link between memory strength and the pupil response. Future studies could extend these findings to other tasks such as visual-word recognition and language production. Pupillometry may also inform computational models of SWR. Just as eye movement research has provided evidence, for example, for the assumption built into TRACE that multiple words partially matching the input simultaneously receive activation as the speech signal unfolds (Allopenna et al., 1998), the pupil response may help inform and refine current models of SWR. For example, the pupil response, an indication of LC-NE system activity, may be linked to the concept of activation implemented into current models of SWR (McClelland and Elman, 1986; Norris, 1994; Hannagan et al., 2013). In TRACE, lexical nodes have a certain base level activation based on a word's occurrence in the language. As the speech signal unfolds, lexical nodes receive activation from sublexical nodes that match the perceptual input. The lexical node that reaches a certain threshold first is selected when its activation level exceeds that of other active nodes by a predetermined value over a certain amount of consecutive time slices. Thus words with higher baseline activation reach the threshold sooner and are recognized earlier compared to words with lower baseline activation. The same mechanism can explain neighborhood effects. Words with more similar sounding neighbors are recognized more slowly because more words compete for selection and thus more perceptual evidence is needed so that target activation exceeds competitor activation. Interactive activation models may also explain the larger neighborhood effect for the less proficient speakers. Less language experience may result in less precise phonological representations, which may be modeled by making competitor inhibition less efficient (c.f. Diependaele et al., 2013). The pupil response may be thought of as reflecting the amount of activation needed for a word to reach threshold. An earlier and lower peak would thus indicate that less activation was needed for a word to be recognized. While further studies are needed to gain a better understanding of the pupil response during lexical retrieval, results from the current and previous studies suggest that pupillometry may have much to offer to further our understanding of SWR. In addition, while the visual-world paradigm has furthered our understanding of the dynamics of lexical competition during SWR (Magnuson et al., 2007), one limitation of the paradigm is that is depends on the presence of visual stimuli (either pictures or printed words). The advantage of measuring the pupil response may be that pictures are not necessarily needed. For example, participants could be aurally presented with a word with a blank screen and then decide whether a later presented picture matched the word or not (c.f. Kuipers and Thierry, 2011). Such a study could also tease apart task effects associated with the visual-world paradigm (e.g., picture-driven language activation) from effects associated with processes of SWR.

## Conclusion

The present study extended previous findings of larger FEs in bilingual and second language speakers in picture naming and visual world recognition (Gollan et al., 2005, 2008, 2011; Duyck et al., 2008; Ivanova and Costa, 2008; Whitford and Titone, 2012; Diependaele et al., 2013) to auditory word recognition. FEs were modulated by language proficiency in the group of bilinguals, suggesting that lexical access in this group may have been delayed because of reduced language experience as a result of later and less frequent exposure to English compared to monolingual speakers. Furthermore, the results from the present study suggest that the bilinguals also experienced more lexical competition during SWR compared to the monolinguals, perhaps because of less precise phonological representations of words in long-term memory (Imai et al., 2005) or reduced sensitivity to acoustic phonetic cues (Bradlow and Pisoni, 1999), which may also have to do with reduced language experience. Taken together, the results reported here showed that bilingualism should be viewed as a continuous rather than categorical variable (c.f. Luk and Bialystok, 2013), with language experience being the modulating factor. In addition, the present results support the hypothesis that the pupil response during the recognition of spoken words reflects retrieval effort (c.f. van Rijn et al., 2012).

### Conflict of interest statement

The author declares that the research was conducted in the absence of any commercial or financial relationships that could be construed as a potential conflict of interest.

## Footnotes

^1^Papesh et al. did not report a pairwise comparison between the high and low frequency condition but the standard errors of the means suggest that the difference was not statistically reliable. Perhaps the number of trials per condition, 20, was not sufficient to find a reliable effect.

^2^Due to experimenter error, the verbal analogies test was not administered to one monolingual participant. Because picture vocabulary scores predicted oral language ability scores well (*R*^2^ = 0.91), this missing value was replaced by the predicted score based on the picture vocabulary test.

^3^Because some pictures from the naming experiment also appeared in the eye-tracking experiment (*k* = 36), whether a picture had been previously named was entered as a control variable in the regression model.

^4^As described in the methods, there were two conditions in which the target pictures were repeated so that they appeared once with a cohort competitor (a referent whose initial sounds overlapped with the target word) and once without. Because each target picture appeared twice, it served as its own control. To test whether the presence of a cohort competitor had an effect on PA or PL, each condition (English competitor/ Spanish competitor) was tested separately. The results showed that the presence of a competitor had no main effect on either of the dependent variables. However, when each group was tested separately, the presence of an English PC had an effect on PLs for the late bilinguals such that the peak dilation occurred 120 ms (*SE* = 45, *p* = 0.0084) later compared to the control condition. Furthermore, the presence of an English PC had an effect on PAs for the monolinguals, with the amplitude being 0.02 mm (*SE* = 0.008, *p* = 0.0149) greater compared to the control condition. Therefore trials with an English PC were excluded from the analysis. Note that including those trials did not change the pattern of results. The presence of a Spanish PC had no effect for any group (all *ps* > 0.28) and therefore those trials were included.Note that the cohort competitor manipulation was not of interest for the present analysis; these trials were only included to achieve greater power. Therefore these results will not be further interpreted.

## Appendix

**Table A1:** **Target word characteristics and distractor pictures used in the experiment**.

**Target picture**	**Log10 subtitle frequency**	**Number of neighbors**	**Number of phonemes**	**Spanish cognate**	**Condition**	**Picture 2**	**Picture 3**	**Picture 4**
Ant	2.44	11	3	0	NoComp	Airplane	Harmonica	Arch
Barn	2.84	17	4	0	NoComp	Light bulb	Hoe	Nose
Bat	3.02	46	3	0	NoComp	Lettuce	Window	Thermos
Bee	2.72	55	2	0	NoComp	French horn	Lion	Groceries
Bench	2.69	10	4	0	NoComp	Frying pan	Turtle	Cutting board
Bow	3.02	58	2	0	NoComp	Beetle	Grill	Lizard
Cactus	2.17	0	6	1	NoComp	Helicopter	Toaster	Jar
Can	5.43	46	3	0	NoComp	Nail	Wineglass	Zipper
Cap	2.98	41	3	0	NoComp	Duck	Saxophone	Swordfish
Car	4.39	43	3	0	NoComp	Accordion	Book	Television
Cat	3.53	43	3	0	NoComp	Baby carriage	Arm	Fishing reel
Corn	2.86	28	4	0	NoComp	Pot	Truck	Doghouse
Cow	3.12	36	2	0	NoComp	Pig	Wagon	Chimney
Cup	3.42	19	3	0	NoComp	House	Spider	Fan
Doll	3.10	26	3	0	NoComp	Basket	Toothbrush	Chest
Eye	3.76	15	1	0	NoComp	Deer	Vest	Rocket
Fan	3.25	36	3	0	NoComp	Leg	Stool	Pyramid
Guitar	2.90	1	5	1	NoComp	Knife	Thimble	Dart
Hand	4.15	17	4	0	NoComp	Star	Shower	Dolphin
Hat	3.52	42	3	0	NoComp	Dresser	Beetle	Stethoscope
Lamb	2.74	37	3	0	NoComp	Cake	Toucan	Tramcar
Lamp	2.82	13	4	1	NoComp	Goggles	Flamingo	Fern
Lemon	2.79	9	5	1	NoComp	Chicken	Strawberry	Propeller
Mouse	2.99	18	3	0	NoComp	Glove	Rooster	Horseshoe
Panda	2.04	5	5	1	NoComp	Record player	Yoyo	Thermometer
Pen	3.10	37	3	0	NoComp	Light switch	Wrench	Igloo
Piano	3.10	0	5	1	NoComp	Lips	Raccoon	Syringe
Pipe	3.00	19	3	1	NoComp	Lantern	Skunk	Whip
Rat	3.22	44	3	1	NoComp	Nut	Shoe	Totem
Safe	3.86	15	3	0	NoComp	Ironing board	Sled	Spider web
Seal	2.88	56	3	0	NoComp	Mountain	Snowman	Jellyfish
Swan	2.54	13	4	0	NoComp	Fly	Rake	Bat
Toe	2.81	59	2	0	NoComp	Cloud	Watch	Anteater
Top	3.83	27	3	0	NoComp	Ear	Rolling pin	Scoop
Train	3.69	12	4	1	NoComp	Bird	Spinning wheel	Parachute
Vase	2.30	24	3	0	NoComp	Pants	Salt shaker	Fishbowl
Well	5.18	40	3	0	NoComp	Potato	Watermelon	Pelican
Alligator	2.25	0	7	1	SpComp	Pliers	Squirrel	Doghouse
Ball	3.73	47	3	0	SpComp	Scale	Ostrich	Fire hydrant
Book	3.96	25	3	0	SpComp	Buffalo	Octopus	Ladybug
Boot	2.76	43	3	1	SpComp	Donkey	Whistle	Shark
Bottle	3.41	16	4	1	SpComp	Sailboat	Spoon	Rope
Brush	2.86	4	4	0	SpComp	Arm	Thumb	Parrot
Bus	3.58	25	3	1	SpComp	Flag	Basin	Vulture
Camera	3.46	0	5	1	SpComp	Bell	Doorknob	Cutting board
Closet	3.14	0	6	0	SpComp	Nail	Tennis racket	Microscope
Cymbals	1.40	3	6	0	SpComp	Belt	Spatula	Peas
Desk	3.35	3	4	0	SpComp	Finger	Swing	Lawnmower
Drum	2.64	9	4	0	SpComp	Camel	Rocking chair	Koala
Eagle	2.77	5	3	0	SpComp	Church	Tie	Ladle
Envelope	2.71	0	7	0	SpComp	Plug	Watering can	Ferris wheel
Flower	3.07	6	4	0	SpComp	Flute	Scorpion	Faucet
Funnel	1.76	8	4	0	SpComp	Skirt	Ruler	Bicycle
Globe	2.43	5	4	0	SpComp	Balloon	Hair	Dragonfly
Gun	4.04	28	3	0	SpComp	Glasses	Colander	Baseball glove
Kettle	2.16	17	4	0	SpComp	Cheese	Feather	Flashlight
Lungs	2.73	10	4	0	SpComp	Lobster	Shirt	Door
Monkey	3.23	5	5	0	SpComp	Apple	Dresser	Hamburger
Moon	3.41	34	3	0	SpComp	Doll	Duck	Worm
Needle	2.79	11	4	0	SpComp	Nest	Snail	Ashtray
Peach	2.51	29	3	0	SpComp	Foot	Paddle	Walrus
Peanut	2.80	0	5	0	SpComp	Pineapple	Heart	Bench
Pumpkin	2.74	1	7	0	SpComp	Bread	Whale	Compass
Telephone	3.22	0	7	1	SpComp	Fork	Net	Nail file
Button	3.16	9	4	1	EngComp	Butterfly	Dress	Cigar
Cage	3.02	17	3	0	EngComp	Cake	Fish	Crown
Candle	2.61	15	5	0	EngComp	Cannon	Football	Dog
Clock	3.48	17	4	0	EngComp	Closet	Fork	Door
Dolphin	2.15	0	6	1	EngComp	Dollar	Hammer	Saw
Feather	2.53	11	4	0	EngComp	Fence	Horse	Screwdriver
Flag	2.95	7	4	0	EngComp	Flashlight	Key	Ashtray
Hammer	2.80	9	4	0	EngComp	Hammock	Owl	Balloon
Ladle	1.59	7	4	0	EngComp	Ladybug	Pear	Banana
Mountain	3.26	2	5	0	EngComp	Mouse	Pencil	Bed
Penguin	2.17	0	7	1	EngComp	Pencil	Tree	Broom
Tire	2.80	31	3	0	EngComp	Tiger	Barrel	Snake
Truck	3.57	6	4	0	EngComp	Trumpet	Sandwich	Helicopter
Watch	4.23	9	3	0	EngComp	Watermelon	Elephant	Pen
Mean	3.04	19.13	3.87	Total: 18				
*SD*	0.69	16.81	1.27					

NoComp, no competitor condition; SpComp, Spanish competitor condition; EngComp, English competitor condition. Targets with a competitor were also presented without a competitor. In this case, the competitor picture was replaced with another competitor from the same condition. For example, mountain appeared paired with mouse (competitor) and with pencil (control). Log10 subtitle frequency was taken from Brysbaert and New (2009). Information about the number of phonological neighbors was taken from Balota et al. (2007). All pictures came from Cycowicz et al. (1997) except for the picture of dollar.
